# GH Response to Glucagon in Transition: Role of Body Mass Index and Etiology in Childhood-onset GH Deficiency

**DOI:** 10.1210/clinem/dgaf674

**Published:** 2026-01-12

**Authors:** Daniela Fava, Stefano Parodi, Alessia Angelelli, Caterina Tedesco, Flavia Napoli, Anna Elsa Maria Allegri, Giuseppa Patti, Roberto Gastaldi, Chiara Santucci, Barbara Vanorio, Claudia Caridi, Nadia Gabriella Maiorano, Rosa Fumo, Marta Panciroli, Alessandro Naim, Alessandro Stefani, Elena Lucia De Rose, Erica Data, Mohamad Maghnie, Natascia Di Iorgi

**Affiliations:** Department of Neuroscience, Rehabilitation, Ophthalmology, Genetics, Maternal and Child Health, University of Genoa, Genoa 16132, Italy; Pediatric Endocrinology Unit, Department of Pediatrics, IRCCS Istituto Giannina Gaslini, Genoa 16147, Italy; Epidemiology and Biostatistics Unit, Scientific Directorate, IRCCS Istituto Giannina Gaslini, Genoa 16147, Italy; Department of Neuroscience, Rehabilitation, Ophthalmology, Genetics, Maternal and Child Health, University of Genoa, Genoa 16132, Italy; Pediatric Endocrinology Unit, Department of Pediatrics, IRCCS Istituto Giannina Gaslini, Genoa 16147, Italy; Pediatric Endocrinology Unit, Department of Pediatrics, IRCCS Istituto Giannina Gaslini, Genoa 16147, Italy; Pediatric Endocrinology Unit, Department of Pediatrics, IRCCS Istituto Giannina Gaslini, Genoa 16147, Italy; Pediatric Endocrinology Unit, Department of Pediatrics, IRCCS Istituto Giannina Gaslini, Genoa 16147, Italy; Department of Neuroscience, Rehabilitation, Ophthalmology, Genetics, Maternal and Child Health, University of Genoa, Genoa 16132, Italy; Pediatric Endocrinology Unit, Department of Pediatrics, IRCCS Istituto Giannina Gaslini, Genoa 16147, Italy; Pediatric Endocrinology Unit, Department of Pediatrics, IRCCS Istituto Giannina Gaslini, Genoa 16147, Italy; Department of Neuroscience, Rehabilitation, Ophthalmology, Genetics, Maternal and Child Health, University of Genoa, Genoa 16132, Italy; Department of Neuroscience, Rehabilitation, Ophthalmology, Genetics, Maternal and Child Health, University of Genoa, Genoa 16132, Italy; Department of Neuroscience, Rehabilitation, Ophthalmology, Genetics, Maternal and Child Health, University of Genoa, Genoa 16132, Italy; Department of Neuroscience, Rehabilitation, Ophthalmology, Genetics, Maternal and Child Health, University of Genoa, Genoa 16132, Italy; Department of Neuroscience, Rehabilitation, Ophthalmology, Genetics, Maternal and Child Health, University of Genoa, Genoa 16132, Italy; Department of Neuroscience, Rehabilitation, Ophthalmology, Genetics, Maternal and Child Health, University of Genoa, Genoa 16132, Italy; Department of Neuroscience, Rehabilitation, Ophthalmology, Genetics, Maternal and Child Health, University of Genoa, Genoa 16132, Italy; Department of Neuroscience, Rehabilitation, Ophthalmology, Genetics, Maternal and Child Health, University of Genoa, Genoa 16132, Italy; Department of Neuroscience, Rehabilitation, Ophthalmology, Genetics, Maternal and Child Health, University of Genoa, Genoa 16132, Italy; Pediatric Endocrinology Unit, Department of Pediatrics, IRCCS Istituto Giannina Gaslini, Genoa 16147, Italy; Department of Neuroscience, Rehabilitation, Ophthalmology, Genetics, Maternal and Child Health, University of Genoa, Genoa 16132, Italy; Pediatric Endocrinology Unit, Department of Pediatrics, IRCCS Istituto Giannina Gaslini, Genoa 16147, Italy; Department of Neuroscience, Rehabilitation, Ophthalmology, Genetics, Maternal and Child Health, University of Genoa, Genoa 16132, Italy; Pediatric Endocrinology Unit, Department of Pediatrics, IRCCS Istituto Giannina Gaslini, Genoa 16147, Italy

**Keywords:** GH deficiency, transition, glucagon, young adults, children cancer survivors, brain tumors, congenital hypopituitarism

## Abstract

**Context:**

The glucagon stimulation test (GST) is increasingly used as an alternative to the insulin tolerance test for diagnosing persistent GH deficiency (GHD) during transition, though its accuracy and appropriate cutoff values are still uncertain.

**Objective:**

To investigate the GH response to GST in transitional-age patients with childhood-onset GHD (CO-GHD), with a focus on the influence of body mass index (BMI) and the underlying etiology.

**Patients and Methods:**

A total of 180 patients with CO-GHD (median age 17.39 years) underwent GST. They were grouped based on the number of pituitary deficiencies and magnetic resonance imaging findings into isolated GHD (n = 80), organic moderate GHD (1-2 deficiencies with congenital or acquired anomalies, n = 63), and organic severe GHD (≥3 deficiencies with complex central nervous system abnormalities, n = 37). Additionally, patients were classified by BMI as normal weight, overweight, or obesity, according to age-appropriate BMI criteria. Childhood cancer survivors (CCS) accounted for 42% of the cohort.

**Results:**

Peak GH response to GST showed a significant inverse association with the severity of pituitary dysfunction (*P* < .001) and an inverse correlation with BMI SD score (ρ = −0.46, *P* < .001). However, adjusting by disease group strongly reduced the impact of BMI on the GST response. When stratified by etiology or CCS status, GH peaks were primarily influenced by hypothalamic-pituitary damage with BMI showing a minimal effect.

**Conclusion:**

The GST provides valuable insights into GH deficiency in transitional-age patients with CO-GHD. GH response is primarily influenced by the severity of pituitary dysfunction, with BMI playing a minimal role once adjusted for etiology.

The transition period between late adolescence and early adulthood is a crucial phase in the management of patients with childhood-onset GH deficiency (CO-GHD) (1-7).

During this stage of life, the reassessment of GH secretion is essential to determine the persistence of the GH deficiency (GHD) and guide long-term treatment decisions. The transition phase is characterized by significant physiological changes in body composition, bone mineral density, and lipid metabolism. All these maturing processes may be impacted by GHD (8-13).

Diagnosing persistent GHD in this age group poses specific challenges, as spontaneous GH secretion declines with age (14, 15), and the criteria for GHD differs from those applied in childhood (16). Among the various stimulation tests available, the glucagon stimulation test (GST) has emerged as a reliable alternative to the “gold standard” insulin tolerance test (ITT), particularly in cases where the ITT is contraindicated (17, 18).

The GST induces GH secretion through hypoglycemia-independent mechanisms, possibly involving central adrenergic pathways, particularly noradrenergic stimulation, as well as other factors such as stress response, fluctuations in glucose and insulin levels, or peptidic fragments of glucagon. However, the exact physiological mechanism remains incompletely understood (17). Despite widespread use of the GST in adult and child populations, its diagnostic accuracy for transitional-age patients remains under ongoing evaluation.

The diagnostic cutoff values of GHD after GST proposed by the 2019 American Association of Clinical Endocrinologists (AACE) guidelines in transition age are based on studies conducted in adults. In these patients, research has shown that cutoffs vary due to differences in assay sensitivity, body mass index (BMI), individual variability in GH secretion, and pretest probability of permanent GHD (19). As it was found that the response to the GST decreases with increasing BMI (20), the AACE guidelines suggest a lower peak GH cut-point of 1 μg/L for patients with obesity (BMI > 30 kg/m^2^) and a cut-point of ≤3 and ≤1 µg/L for patients with overweight (BMI 25-30 kg/m^2^) and a high and low pretest probability, respectively (19). Recently, in a cohort of 97 CO-GHD patients with a median age of 17.39 years who underwent ITT and GST, we found a high consistency from the GH peak measures after ITT and GST. The results of receiver operating characteristic curve analysis of GST showed that a GH peak value cutoff of 5.8 μg/L was able to correctly classify 91.4% of patients with a high pretest probability of permanent GHD (95% confidence interval 3.16-7.39; sensitivity, 96.0%, specificity 80.0%, positive predictive value 92.3%, negative predictive value 88.9%) (21). We found that BMI SD score (SDS) was predictive of GH response to GST in univariable regression analyses but not in multivariable models (21). The correlation between BMI SDS and GH levels in our cohort appeared to be mainly driven by the significant correlation between BMI SDS and the severity of GHD, but the limited sample size prevented reliable conclusions from being drawn (21).

Hence, this study aimed to determine the diagnostic accuracy of GST in a broader cohort of young adults with CO-GHD, stratified by underlying etiology and BMI.

## Methods

### Patients

This retrospective cohort study included 180 patients (112 males, 68 females) with CO-GHD who underwent a GST (1 mg I.M.) during the transition phase from September 2013 to April 2024 in a single tertiary level academic center (Pediatric Endocrine Unit, IRCCS Istituto Giannina Gaslini, University of Genova; Genova, Italy).

The diagnosis of GHD in childhood or adolescence was established following confirmation of inadequate GH secretion through 2 separate stimulation tests performed on 2 different days (peak GH concentration after ITT, arginine, GST below 10 µg/L until 2014 and less than 8 µg/L thereafter), according to national recommendations (22).

At the time of retesting, 114 out of 180 patients (63.3%) were aged 18 years or younger, while 66 patients (36.7%) were older than 18 years. All patients had achieved adult height, defined by a growth velocity of less than 2 cm/year, and had reached advanced pubertal development (Tanner stage 4-5). Additionally, all patients had discontinued recombinant GH therapy for at least 1 month prior to retesting.

**Figure 1.**
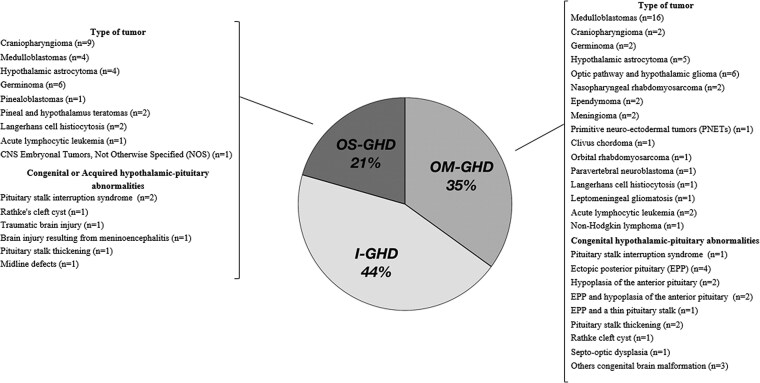
Etiological classification of 180 patients with CO-GHD at the time of retesting. Patients were grouped based on pituitary MRI findings and the number of associated hormone deficiencies into isolated I-GHD, OM-GHD (1-2 deficiencies), and organic severe GHD (≥3 deficiencies with complex central nervous system abnormalities). Abbreviations: CO-GHD, childhood-onset GH deficiency; I-GHD, isolated GH deficiency; MRI, magnetic resonance imaging; OM-GHD, organic moderate GH deficiency; OS-GHD, organic severe GH deficiency.

Following the 2019 AACE guidelines (19) and brain magnetic resonance imaging (MRI) findings at the time of GHD diagnosis, we classified patients into 3 groups (Fig. 1): patients with isolated GHD, normal MRI findings and no history of cancer, were considered idiopathic GHD (I-GHD); patients with 1 or 2 pituitary hormone defects and with congenital anomalies (pituitary stalk interruption syndrome and other cerebral midline defects) and organic hypothalamic-pituitary disease (namely, acquired following brain surgery, craniospinal total body irradiation, or infiltrative disease of the hypothalamus and pituitary stalk) were defined as organic moderate GHD (OM-GHD); and patients with 3 or more pituitary hormone deficiencies and with congenital or acquired anomalies involving the hypothalamic-pituitary region or a central nervous system tumor were classified as organic severe GHD (OS-GHD).

At the time of retesting, all patients with hormone deficiencies were receiving replacement therapy as needed, according to current recommendations (23).

Patients were categorized into weight-status groups using age-appropriate criteria. For individuals under 18 years, BMI classification followed World Health Organization BMI-for-age standards: normal weight (≤ + 1 SDS), overweight (> + 1 and < + 2 SDS), and obesity (≥ + 2 SDS) (24). For those age 18 years or older, classification was based on absolute BMI values according to World Health Organization adult definitions: normal weight (<25 kg/m^2^), overweight (25-29.9 kg/m^2^), and obesity (≥30 kg/m^2^) (25). This allowed for consistent categorization across the cohort.

The study was approved by the ethics committee (Genoa, Italy; protocol number 13777/21), and written informed consent was obtained from parents or caregiver of patients after full explanation of the study according to the Declaration of Helsinki.

### Data Collection

We retrospectively collected clinical and treatment data (date of birth, cancer diagnosis, oncological treatments, date of GHD diagnosis and of additional pituitary defects, date of GH retesting), anthropometric parameters based on Tanner charts (26-28) (height SDS, pubertal Tanner stage), and biochemical data including baseline IGF-1. BMI SDS was calculated according to Cole et al (29).

All these data were recorded at the time of GHD diagnosis (Table 1) and at retesting (Table 2). At GHD diagnosis, all subjects included in the study underwent brain MRI examination and a high-resolution sellar MRI, which provided detailed information of the suprasellar compartment and the pituitary stalk. Bone age (BA) at diagnosis was assessed according to Greulich and Pyle (30) and reevaluated by the same expert endocrinologist (D.F.) at the time of the study. The delta BA for chronological age (ΔBA/CA) was calculated. Parental height measurements, when available, were used to calculate target height SDS (mean of parental height/2 ± 6.5 cm) and delta target height SDS; IGF-1 was expressed in SDS based on normative data (31, 32).

**Table 1.** Characteristics of 180 patients with childhood-onset GHD at the time of diagnosis.

Variable	Study population	Idiopathic-GHD	OM-GHD	OS-GHD	*P*-value A	Congenital-GHD	Acquired-GHD	*P*-value B
Clinical characteristics								
No. of patients (males/females)	180 (112/68)	80 (54/26)	63 (39/24)	37 (19/18)	0.245	19 (13/6)	81 (45/36)	0.248
Age (years)*	11.56 (8.59–13.67)	11.48 (8.34–13.51)	11.48 (8.62–14.48)	11.66 (8.91–13.04)	0.567	9.72 (3.09–12.53)	11.73 (9.65–14.05)	0.010
Height SDS**	−1.98 (−2.43–−1.05)	−2.38 (−2.73–−2.01)	−1.60 (−2.34–0.43)	−0.75 (−1.61–−0.23)	<0.001	−2.34 (−2.85–−1.93)	−1.13 (−1.79–−0.27)	<0.001
BMI SDS***	0.55 (−0.60–1.84)	−0.32 (−0.98–1.06)	0.60 (−0.17–1.60)	2.08 (0.81–2.54)	<0.001	−0.04 (−1.90–0.55)	1.29 (0.48–2.36)	<0.001
Delta H–TH SDS (H SDS – TH SDS) ^§^	−1.18 (−1.94–−0.49)	−1.41 (−2.04–−1.01)	−0.77 (−1.46–0.19)	−1.08 (−2.06–0.11)	0.003	−1.46 (−1.93–−0.62)	−0.81 (−1.66–−0.29)	<0.001
Delta CA–BA^§§^	−1.09 (−2.07–−0.12)	−1.64 (−2.53–−0.93)	−0.59 (−1.38–−0.01)	−0.40 (−1.75–0.14)	<0.001	−1.38 (−2.37–−0.94)	−0.34 (−1.23–0.06)	<0.001
Peak GH provocative tests								
Arginine (μg/L) (n=146)	4.06 (1.91–6.21)	5.62 (3.29–6.93)	3.96 (1.78–5.99)	0.60 (0.10–3.46)	<0.001	2.70 (1.80–5.40)	2.54 (0.65–4.70)	<0.001
ITT (μg/L) (n=114)	3.46 (1.73–4.88)	4.44 (3.27–5.71)	2.73 (1.57–3.84)	0.96 (0.31–1.90)	<0.001	2.68 (1.90–4.46)	1.60 (0.69–3.29)	<0.001
GST (μg/L) (n=35)	2.77 (0.81–4.26)	4.21 (3.10–7.00)	1.97 (0.81–4.08)	1.23 (0.44–2.66)	0.032	3.25 (2.19–4.28)	1.14 (0.60–2.91)	0.025
Biochemical data								
IGF-1 SDS (n=142)	−1.96 (−2.99–−1.00)	−1.56 (−2.44–−0.90)	−2.07 (−2.82–−1.00)	−3.20 (−3.84–−1.69)	<0.001	−2.13 (−6.24–−0.40)	−2.48 (−3.49–−1.37)	0.007

Data are reported as median (IQR). *P*-value A refers to the comparison among Idiopathic-GHD, OM-GHD, and OS-GHD. *P*-value B refers to the comparison among Idiopathic-GHD, Congenital-GHD, and Acquired-GHD.

Abbreviations: BA, bone age; CA, chronological age; GHD, growth hormone deficiency; GST, glucagon stimulation test; ITT, insulin tolerance test; OM-GHD, organic moderate GHD; OS-GHD, organic severe GHD; SDS, standard deviation score; TH, target height.

*3 missing values; ** 27 missing values; *** 31 missing values; ^§^ 33 missing values; ^§§^ 62 missing.

**Table 2.** Characteristics of 180 patients with childhood-onset GHD at the time of retesting.

Variable	Study population (N=180)	Idiopathic-GHD (N=80)	OM-GHD (N=63)	OS-GHD (N=37)	*P*-value A	Congenital-GHD (N=19)	Acquired-GHD (N=81)	*P*-value B
Clinical characteristics								
Age (years)	17.39 (15.98 - 18.62)	17.47 (16.30 - 18.48)	17.57 (15.65 - 18.40)	17.23 (15.97 - 18.98)	0.825	17.29 (16.03 - 18.18)	17.32 (15.65 - 19.00)	0.812
Height SDS	−1.19 (−1.72 - −0.18)	−1.23 (−1.71 - −0.39)	−1.29 (−2.24 - −0.36)	−0.62 (−1.5 - 0.69)	0.005	−0.69 (−2.01 - 0.42)	−1.19 (−1.69 - 0.20)	0.421
BMI SDS	0.41 (−0.35 - 1.49)	0.0 (−0.91 - 0.71)	0.41 (−0.10 - 1.60)	1.60 (0.70 - 2.37)	< 0.001	0.40 (−0.40 - 1.60)	1.15 (0.10 - 1.85)	< 0.001
Peak GH on provocative test								
ITT (µg/L)*	6.19 (1.85 - 15.03)	13.60 (9.12 - 18.22)	3.22 (1.60 - 5.72)	0.58 (0.23 - 1.81)	< 0.001	4.64 (2.18 - 10.78)	1.71 (0.60 - 3.70)	< 0.001
GST (µg/L)	5.86 (1.37 - 12.24)	12.00 (9.83 - 21.00)	3.07 (1.14 - 5.33)	0.62 (0.20 - 1.37)	< 0.001	5.32 (0.64 - 9.10)	1.38 (0.62 - 3.21)	< 0.001
Biochemical data								
Baseline blood glucose (mg/dl)	89.5 (85.0 - 95.0)	92.0 (87.5 - 95.5)	90.0 (86.0 - 96.0)	81.0 (78.0 - 87.0)	< 0.001	88.0 (84.0 - 97.0)	87.0 (81.0 - 93.0)	0.004
IGF-1 SDS (n=176)	−0.86 (−2.43 - −0.04)	−0.04 (−0.59 - 0.91)	−1.76 (−2.82 - −0.61)	−2.76 (−3.74 - −2.19)	< 0.001	−1.18 (−2.98 - −0.46)	−2.31 (−3.23 - −1.17)	< 0.001

Data are reported as median (IQR). *P*-value A refers to the comparison among Idiopathic-GHD, OM-GHD, and OS-GHD. *P*-value B refers to the comparison among Idiopathic-GHD, Congenital-GHD, and Acquired-GHD.

Abbreviations: BA, bone age; CA, chronological age; GHD, growth hormone deficiency; GST, glucagon stimulation test; ITT, insulin tolerance test; OM-GHD, organic moderate GHD; OS-GHD, organic severe GHD; SDS, standard deviation score; TH, target height.

*51 missing values.

### Biochemical Evaluation

The GST started between 8.00 and 9.00 Am, after an overnight fasting at the dose of 1 mg intramuscular. Blood samples were obtained at 0, 30, 60, 90, 120, 150, and 180 minutes after glucagon administration through an IV heparin-locked line for the determination of GH and blood glucose; cortisol levels were measured at 0, 120, 150, and 180 minutes. All samples were analyzed together immediately after test completion, without storage; results were available the same day.

Serum GH was measured by chemiluminescent immunometric assay (Immulite 2000, GH; Diagnostic Products Corporation, Los Angeles, CA; international reference preparation 98/574). The inter- and intra-assay coefficients of variation were 4.2% to 6.6% and 2.9% to 4.6%, respectively, at GH concentrations of 2.6 to 17 µg/L. All serum IGF-I samples were measured by chemiluminescent immunometric assay (Immulite 2000; Diagnostic Products Corporation). The intra- and interassay coefficients of variation were 3.4% and 7.1%, respectively, and the sensitivity of the method was 2.6 nmol/L. After centrifugation at 4 °C, plasma was separated and stored at −20 °C. Serum glucose was measured automatically with a hexokinase-catalyzed glucose oxidase method.

In patients on testosterone replacement, the GST was performed at least 2 weeks after the last testosterone enanthate injection or 4 weeks after the last testosterone undecanoate injection, to minimize the acute effect of sex steroids on GH response.

### Statistical Methods

Absolute frequencies and percentages were calculated to describe qualitative variables. Mean and SD were reported for normally distributed quantitative variables, while median and interquartile range were used in the presence of nonnormal distributions. Normality was assessed by visual inspection of the related histograms and quantile-quantile plots. Between-group comparisons in normally distributed variables were made by the Student's *t*-test and 1-way ANOVA, while the Mann–Whitney U-test and the Kruskal–Wallis test were employed for nonnormal variables. The association between continuous variables was assessed using the nonparametric Spearman's ρ correlation coefficient, given the nonnormal distribution of some variables. A multivariable linear regression model was used to evaluate the association between the GH peak to GST, BMI SDS, and the disease classification (idiopathic, congenital, and acquired GHD) after normalizing the marker distribution by the natural logarithm transformation. Analysis of residuals was performed to check the violation of the regression assumptions. All analyses were carried out by STATA/MP for Windows statistical package (18th release, STATA Corp LLC, College Station, TX, 2023).

## Results

Eighty patients (44.4%) of the cohort belonged to the I-GHD group; these patients were considered likely to have a low pretest probability for permanent GHD. Sixty-three patients (35%) belonged to the OM-GHD group. This group comprised 15 subjects (23.8%) with congenital anomalies involving the hypothalamic-pituitary region (pituitary stalk interruption syndrome and other cerebral midline defects) and 48 subjects (76.2%) survived from childhood cancer [childhood cancer survivors (CCS)] (Fig. 1). These patients were considered likely to have a high pretest probability for permanent GHD.

Thirty-seven patients (20.6%) belonged to the OS-GHD group. This group included 7 subjects (18.9%) with congenital or acquired anomalies of the hypothalamic-pituitary region and 30 CCS subjects. Reassessing GH secretion in these patients would not have been necessary under current recommendations because of their high risk of permanent GHD (33).

In our cohort, 19 patients (10.5%) had GHD resulting from congenital brain abnormalities. These patients were identified as belonging to the congenital GHD group. Fifteen patients in this group were part of the OM-GHD group and 4 patients were part of the OS-GHD group (Fig. 1). Eighty-one patients (44.7%) had acquired brain abnormalities. These patients were identified as belonging to the acquired GHD group. Forty-eight patients of this group were part of the OM-GHD group and 33 patients were part of the OS-GHD group (Fig. 1). Seventy-six patients of this group (93.8%) were CCS.

Twenty-six subjects of the OM-GHD patients had an isolated GHD, and 37 subjects had a second hypothalamic-pituitary defect (n = 19 had TSH deficiency, n = 5 had ACTH deficiency, n = 10 had hypogonadotropic hypogonadism, n = 3 had vasopressin deficiency). Among the OS-GHD patients, 7 had panhypopituitarism, 12 had 4 pituitary defects including GHD, and 18 had 3 pituitary defects.

At the time of retesting, all patients with hormone deficiencies were receiving replacement therapy with hydrocortisone (7-9 mg/m^2^/day) and/or L-thyroxine and/or desmopressin acetate (range 30-120 mcg 3 times a day orally), as needed.

Thirteen male patients in the entire cohort had hypogonadism (n = 4 in the OM-GHD group, n = 9 in the OS-GHD group). They were receiving testosterone intramuscularly (testosterone enanthate 200-250 mg/every 3 weeks or testosterone undecanoate 1000 mg/every 12 weeks). Seventeen females had hypogonadism (n = 6 in the OM-GHD group, n = 11 in the OS-GHD group). Thirteen of them were on transdermal hormone replacement therapy (n = 5 in the OM-GHD group, n = 8 in the OS-GHD group), consisting of 17β-estradiol 50 mcg/day and oral medroxyprogesterone acetate (10 mg/day) for 11 days a month; 5 females were on oral estroprogestin replacement therapy (INN-nomegestrol acetate 2.5 mg/estradiol 1.5 mg for 24 days a month).

### Clinical and Biochemical Characteristics of the Study Cohort at CO-GHD Diagnosis

The clinical characteristics, GH peak and IGF-1 SDS values at first GHD diagnosis for each group of patients are reported in Table 1. No statistically significant difference emerged between the age at GHD diagnosis in the I-GHD, OM-GHD, and OS-GHD groups. Patients in the I-GHD group were significantly shorter (*P* < .001), even compared with their target height (*P* = .003). They had a lower BMI SDS and a more delayed BA than patients in the other groups (*P* < .001). Patients in the OS-GHD group showed significantly lower peak GH values during provocative testing compared to other groups (*P* = .032); similarly, IGF-1 SDS values were also significantly reduced in this group (*P* < .001). A comparison of patients in the I-GHD group to those with congenital and acquired GHD revealed a significantly younger age in the congenital GHD cohort (*P* = .010). The acquired GHD group showed greater height SDS, a smaller difference between height SDS and target height SDS, higher BMI SDS, and a smaller gap between BA and chronological age (*P* < .001). Peak GH levels during testing were significantly lower in the acquired GHD group (*P* = .025), along with lower IGF-1 SDS values (*P* = .007).

Among the 149 patients with available BMI data at diagnosis (64 with I-GHD, 52 classified as OM-GHD, and 33 as OS-GHD), 37 individuals (24.8%) were found to have obesity. The prevalence of obesity within each group was 10.9% (n = 7) in the I-GHD group, 23.1% (n = 12) in the OM-GHD group, and 54.5% (n = 18) in the OS-GHD group.

### Clinical Characteristics of the Study Cohort at GHD Retesting

**Figure 2.**
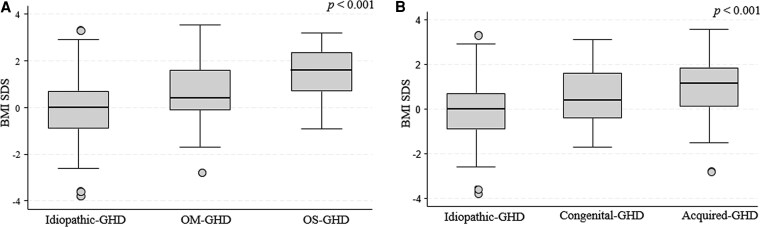
Distribution of BMI SDS across etiological subgroups in CO-GHD patients at the time of GH retesting. (A) Comparison among I-GHD, OM-GHD, and OS-GHD groups (*P* < .001). (B) Comparison among idiopathic, congenital, and acquired GH deficiency groups (*P* < .001). Abbreviations: BMI, body mass index; CO-GHD, childhood-onset GH deficiency; I-GHD, isolated GH deficiency; MRI, magnetic resonance imaging; OM-GHD, organic moderate GH deficiency; OS-GHD, organic severe GH deficiency; SDS, SD score.

The clinical characteristics, GH peak, and IGF-1 SDS values at GHD retesting for each group of patients are reported in Table 2. The median age at GHD retesting was 17.39 years [interquartile range (IQR) 15.97; 18.61], with no statistically significant differences between the groups. Patients in the OS-GHD group were the tallest (*P* = 0.005) and had the highest BMI SDS (*P* < .001) (Table 2 and Fig. 2A). Comparison of the I-GHD group with the acquired and congenital groups showed that the acquired group had significantly higher BMI SDS (*P <* .001) (Table 2 and Fig. 2B).

At GHD retesting, 119 (66.1%) of the entire cohort were of normal weight, 37 (20.6%) were overweight, and 24 (13.3%) had obesity. Among patients with I-GHD (n = 80), the majority were of normal weight (67 patients, 83.8%), while 9 patients (11.2%) were overweight and 4 (5.0%) had obesity. In the OM-GHD group (n = 63), 39 patients (61.9%) had normal weight, 17 (27.0%) were overweight, and 7 (11.1%) had obesity. In contrast, within the OS-GHD group (n = 37), only 13 patients (35.1%) had normal weight, whereas 11 (29.7%) were overweight and 13 (35.1%) had obesity.

When patients were grouped by GHD etiology, 38 out of 81 patients with acquired GHD (46.9%) had normal weight, 25 (30.9%) were overweight, and 18 (22.2%) had obesity. Among those with congenital GHD (n = 19), 14 patients (73.7%) had normal weight, 3 (15.8%) were overweight, and 2 (10.5%) had obesity.

A reduction in obesity prevalence was observed from diagnosis to retesting: 24.8% (37/149) of patients with available BMI data had obesity at diagnosis, compared to 13.3% (24/180) at retesting. Of these 24 patients, 19 already had obesity at the time of diagnosis, while 4 developed obesity during follow-up. Notably, 3 of these 4 patients (1 patient in the I-GHD group and 2 in the OS-GHD group) had previously been overweight, and for 1 patient, BMI data at diagnosis were not available.

Among the patients who had obesity at diagnosis, a reduction in obesity prevalence was observed across all groups. Specifically, only 2 out of 7 patients (28.6%) in the I-GHD group remained obese at retesting, compared to 7 out of 12 patients (58.3%) in the OM-GHD group and 10 out of 18 patients (55.6%) in the OS-GHD group.

### Biochemical Characteristics of the Study Cohort at GHD Retesting

**Figure 3.**
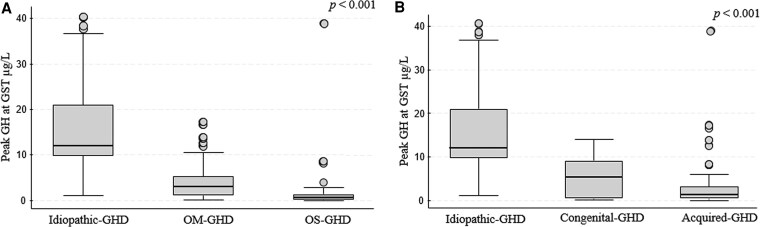
Peak GH response to glucagon stimulation according to GHD etiology. (A) Comparison among I-GHD, OM-GHD, and OS-GHD groups (*P* < .001). (B) Comparison among idiopathic, congenital, and acquired GHD groups (*P* < .001). Abbreviations: GHD, GH deficiency; I-GHD, isolated GH deficiency; OM-GHD, organic moderate GH deficiency; OS-GHD, organic severe GH deficiency.

The median GH peak to GST in the whole cohort was 5.86 μg/L (IQR 1.37; 12.24) (Table 2). In addition, at the first GHD diagnosis, patients in OS-GHD group showed lower peak GH at retesting (0.62 μg/L; IQR 0.20; 1.37) compared to the OM-GHD (3.07 μg/L; IQR 1.14; 5.33) and I-GHD group (12.00 μg/L; IQR 9.83; 21.0) (*P* < .001) (Table 2 and Fig. 3A).

Mean baseline blood glucose in the whole cohort was 89.6 ± 8.6 mg/dL. It was significantly lower in the OS-GHD group (82.1 ± 7.4 mg/dL) compared to the other groups (91.6 ± 9.4 mg/dL in the OM-GHD group and 91.5 ± 6.45 mg/dL in the idiopathic GHD group, *P* < .001). A statistically significant difference in mean basal blood glucose between groups was also found considering subjects with acquired brain lesions (86.9 ± 9.2 vs 91.3 ± 7.9 in patients without brain tumors, *P* < .001) and those with acquired and congenital abnormalities vs idiopathic patients (87.8 ± 10.1, 89.7 ± 8.7, and 91.5 ± 6.5, respectively, *P* = .021).

Information on IGF-1 SDS was available for 176 patients. The median IGF-1 SDS in the whole cohort was −0.86 (IQR −2.43; −0.04). IGF-1 SDS was lower in the OS-GHD group (−2.76; IQR −3.74; −2.19) compared to the OM-GHD (−1.76; IQR −2.82; −0.61) and I-GHD group (−0.04, IQR −0.59; −0.91, *P* < .001).

The acquired GHD group had significantly lower GH peaks (1.38 μg/L; IQR 0.62; 3.21) compared to the congenital GHD (5.32 μg/L; IQR 0.64; 9.10) and I-GHD group (12.00 μg/L; IQR 9.83; 21.0) (*P* < .001, Table 2 and Fig. 3B). The acquired GHD group also had significantly lower IGF-1 SDS values (−2.31; IQR −3.23; −1.17) compared to the congenital GHD (−1.18; IQR −2.98; −0.46) and I-GHD group (−0.04, IQR −0.59; −0.91, *P* < .001, Table 2).

Applying a GH cutoff value of 5.8 µg/L in the study cohort as a threshold value to diagnose GHD in the transition age, GH deficiency was identified in 90 (50.0%) of the patients (Table 3). The highest percentage of those who had a GH peak <5.8 µg/L was found in the OS-GHD group (91.9%), compared to the OM-GHD (77.8%) and I-GHD groups (8.8%, *P* < .001). Similarly, subjects in the acquired GHD group were those in whom GHD was confirmed more frequently (88.9%) than subjects in the congenital (57.9%) and I-GHD groups (8.8%, *P* < .001).

**Table 3.** Distribution of study population by GST test result (<5.8 vs. ≥5.8 µg/L) and GHD etiology.

GH peak (µg/L)	Study population N=180	Idiopathic GHD N=80	Organic moderate GHD N=63	Organic severe GHD N=37	*P*-value A	Congenital GHD N=19	Acquired GHD N=81	*P*-value B
<5.8	90 (50.0%)	7 (8.8%)	49 (77.8%)	34 (91.9%)	<0.001	11 (57.9%)	72 (88.9%)	<0.001
≥5.8	90 (50.0%)	73 (91.3%)	14 (22.2%)	3 (8.1%)		8 (42.1%)	9 (11.1%)	

*P*-value A refers to the comparison among Idiopathic-GHD, OM-GHD, and OS-GHD. *P*-value B refers to the comparison among Idiopathic-GHD, Congenital-GHD, and Acquired-GHD.

Abbreviations: GHD, growth hormone deficiency; OM-GHD, organic moderate GHD; OS-GHD, organic severe GHD.

Among the 90 patients with a peak GH value <5.8 µg/L, 45 (50.0%) were of normal weight, 24 (26.7%) were overweight, and 21 (23.3%) had obesity (Table 4). Among those with obesity, 13 patients had IGF-1 levels below −2 SDS, 10 of whom belonged to the OS-GHD group (Table 5). In contrast, among the 90 patients with a normal GH response to the GST (≥5.8 µg/L), 74 (82.3%) were of normal weight, 13 (14.4%) were overweight, and only 3 (3.3%) had obesity (Table 4).

**Table 4.** Distribution of Study Population by GST Test Results (<5.8 vs. ≥5.8 µg/L) and BMI categories.

BMI	Study population N=180	GH peak (µg/L) ≥5.8 N=90	GH peak (µg/L) <5.8 N=90
Normal weight	119 (66.1%)	74 (82.2%)	45 (50.0%)
Overweight	37 (20.6%)	13 (14.4%)	24 (26.7%)
Obesity	24 (13.3%)	3 (3.3%)	21 (23.3%)

**Table 5.** Distribution of patients based on GH cut-offs, IGF-I SDS values and BMI categories.

GHD etiology	Cut-off GH (µg/L)	Normal weight			Overweight			Obesity		
		IGF-I<-2 SDS (n)	IGF-I≥-2 and <0 SDS (n)	IGF-I≥0 SDS (n)	IGF-I<-2 SDS (n)	IGF-I≥-2 and <0 SDS (n)	IGF-I≥0 SDS (n)	IGF-I<-2 SDS (n)	IGF-I≥-2 and <0 SDS (n)	IGF-I≥0 SDS (n)
Idiopathic-GHD	< 5.8	1	3	2	0	0	0	0	1	0
	≥ 5.8	0	29	30	0	6	2	0	1	2
Organic-M GHD	< 5.8	18	9	1	6	8	0	3	3	1
	≥ 5.8	0	9	2	0	2	1	0	0	0
Organic-S GHD	< 5.8	10	1	0	8	1	0	10	3	0
	≥ 5.8	0	1	1	1	0	0	0	0	0
Congenital-GHD	< 5.8	5	2	0	1	0	0	1	1	0
	≥ 5.8	0	7	0	0	0	1	0	0	0
Acquired-GHD	< 5.8	23	8	1	14	8	0	12	5	1
	≥ 5.8	0	3	3	1	2	0	0	0	0

Missing values: Idiopathic-GHD group (≥5.8 µg/L), n=3; Organic-S GHD group (<5.8 µg/L), n=1; Congenital-GHD group (<5.8 µg/L), n=1.

Abbreviations: GHD, growth hormone deficiency; IGF-I, insulin-like growth factor 1; OM-GHD, organic moderate GHD; OS-GHD, organic severe GHD; SDS, standard deviation score.

**Figure 4.**
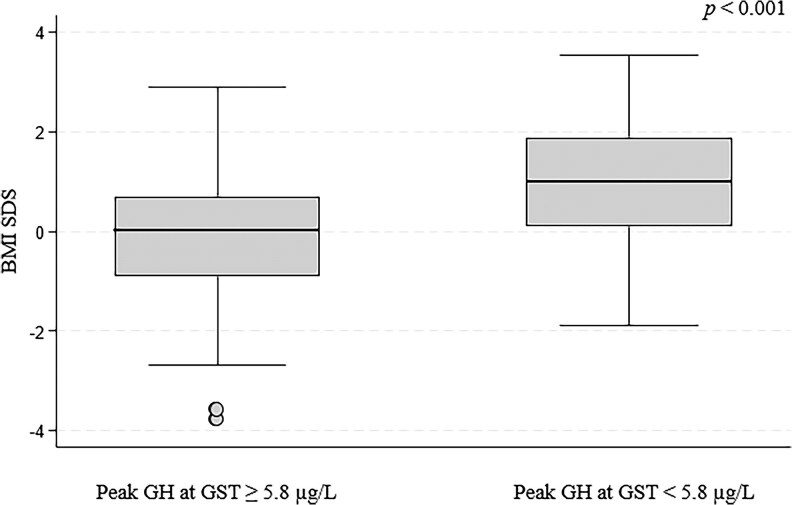
Distribution of BMI SDS based on GH peak response to a glucagon stimulation test using a 5.8 µg/L cutoff. Patients with a GH peak ≥5.8 µg/L had significantly lower BMI SDS compared to those with GH peak <5.8 µg/L (*P* < .001). Abbreviations: BMI, body mass index; SDS, SD score.

Median BMI SDS was significantly higher in patients with peak GH value < 5.8 µg/L at GST compared to patients with peak GH value ≥ 5.8 µg/L (0.99; IQR 0.1; 1.89 vs 0.01; IQR −0.9; 0.7) (*P* < .001, Fig. 4).

Of the 90 patients with a GH peak < 5.8 µg/L, IGF-1 SDS values were available for 89. Among them, 56 (62.9%) had IGF-1 SDS < −2, and 85 (95.5%) had IGF-1 SDS < 0.

### CCS

Seventy-six patients of the entire cohort (42%) were CCS; 46 were in the OM-GHD group and 30 in the OS-GHD group. Sixty-seven (37%) had brain tumors (38 in the OM-GHD and 29 in the OS-GHD group).

The most frequently found brain tumor in our cohort was medulloblastoma (n = 20), followed by craniopharyngioma (n = 11) and pilocytic astrocytoma (n = 9).

Sixty received radiotherapy (38 in the OM-GHD group and 22 in the OS-GHD group), 56 patients underwent cranial o cranial-spinal irradiation (35 in the OM-GHD group, 21 in the OS-GHD group), and 4 received total body irradiation (3 patients with acute lymphoblastic leukemia, 1 patient with non-Hodgkin lymphoma). The median time between radiotherapy stop and GHD retesting was 9.65 years (IQR 6.60;12.39).

Among the 76 CCS in the cohort, 37 (48.7%) were classified as having normal weight, 23 (30.3%) as overweight, and 16 (21.1%) as having obesity. Of those with normal weight, the majority (26 patients, 70.3%) belonged to the OM-GHD group, while the remaining 11 (29.7%) were part of the OS-GHD group.

Among the overweight patients, 14 (60.9%) were in the OM-GHD group and 9 (39.1%) in the OS-GHD group. Similarly, among patients with obesity, 6 (37.5%) were in the OM-GHD group and 10 (62.5%) were classified as having OS-GHD.

Among the 149 patients with available BMI data at diagnosis, CCS accounted for 67.6% (25 out of 37) of those with obesity. Notably, CCS represented most patients with obesity within the organic GHD groups: 83.3% (10 out of 12 patients) in the OM-GHD group and 83.3% (15 out of 18) in the OS-GHD group.

At retesting, 16 of the 24 patients with obesity (66.7%) were CCS. CCS accounted for most patients with obesity within the organic GHD subgroups: 85.7% in the OM-GHD group (6 out of 7 patients) and 77.0% in the OS-GHD group (10 out of 13). A history of radiotherapy exposure was present in all CCS with obesity in the OM-GHD group and in 30.0% of those in the OS-GHD group.

Interestingly, both patients from the OS-GHD group who developed obesity during follow-up, despite a normal BMI at diagnosis, were CCS, and 1 of them had received radiotherapy.

The median GH peak to GST in CCS patients was 1.38 μg/L (IQR 0.61; 3.19). In patients with brain tumors, the median GH peak to GST was 1.33 μg/L (IQR 0.60; 2.64), markedly lower compared to the rest of the cohort (10.62 μg/L; IQR 5.67; 15.9) (*P* < .001). In CCS patients within the OS-GHD group, the GH peak was significantly lower compared to CCS patients in the OM-GHD group (*P* < .001).

### Biochemical Characteristics During the Glucagon Test

**Figure 5.**
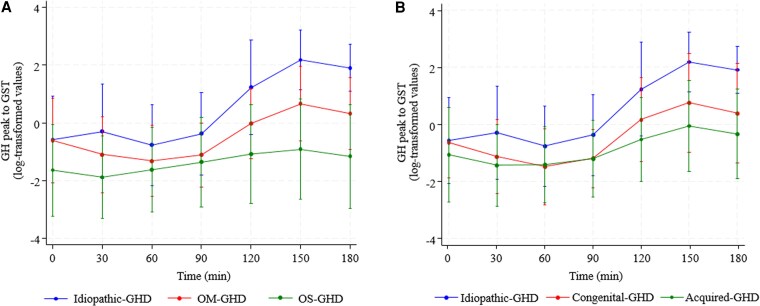
Temporal profile of GH secretion during the glucagon stimulation test, stratified by GHD etiology. (A) Comparison among I-GHD, OM-GHD, and OS-GHD groups. (B) Comparison among idiopathic, congenital, and acquired GHD groups. GH values are presented as log-transformed. Abbreviations: GHD, GH deficiency; I-GHD, isolated GH deficiency; OM-GHD, organic moderate GH deficiency; OS-GHD, organic severe GH deficiency.

GH levels reached their peak at 150 minutes after glucagon injection (mean value 7.4 ± 8.5 mg/dL), while the lowest value was observed after 60 minutes (0.78 ± 1.3 mg/dL). The analysis by group of patients, defined based on GHD characteristics at diagnosis, is shown in Fig. 5, where data are expressed on a logarithmic scale to normalize the values distribution (panel A: I-GHD vs OM-GHD and OS-GHD; panel B: I-GHD vs congenital GHD and acquired GHD). In all considered groups, GH levels were nearly stable between 0 and 90 minutes and increased thereafter, reaching a peak at 150 minutes (Fig. 5A and 5B). In detail, the mean values at the observed peak was 12.7 ± 8.9 mg/dL in I-GHD patients, 3.74 ± 4.28 mg/dL in OM-GHD, 1.92 ± 5.81 mg/dL in OS-GHD, 4.83 ± 4.33 in congenital GHD, and 2.68 ± 5.02 in acquired GHD. In I-GHD and OM-GHD, the lowest mean value was observed at 60 minutes (1.11 ± 1.53, and 0.56 ± 1.1 mg/dL, respectively), in OS-GHD at 30 minutes (0.29 ± 0.34 mg/dL), in congenital GHD at 90 minutes (0.49 ± 0.58 mg/dL), and in acquired GHD at 60 minutes 0.47 ± 0.63 mg/dL.

At each observed time after glucagon injection, OS-GHD patients showed lower values of GH compared with I-GHD and OM-GHD. When non-I-GHD patients were split into congenital GHD and acquired GHD, similar statistically significant differences were found, with patients with an acquired deficit showing the lowest values at any time except for 60 and 90 minutes after glucagon injections, where values were almost equal to those observed among patients with congenital GHD (Fig. 5B).

Blood glucose peaked at 30 minutes postglucagon (mean 142.8 ± 22.3 mg/dL) across all groups: 148.1 ± 20.4 in I-GHD, 140.2 ± 23.3 in OM-GHD, and 136.2 ± 22.6 mg/dL in OS-GHD. The lowest mean values occurred at 150 minutes (total 73.9 ± 10.0 mg/dL; OM-GHD 75.1 ± 10.4; OS-GHD 69.7 ± 9.7 mg/dL). Similarly, in congenital and acquired GHD, glucose peaked at 30 minutes and reached nadir at 150 minutes, whereas in I-GHD it declined earlier, with a minimum of 73.1 ± 12.7 mg/dL at 120 minutes.

I-GHD patients showed the most dynamic glycemic trend, with an early peak, sharper decline, and secondary rise, whereas OS-GHD patients had the lowest nadir and slower recovery. These findings support a biphasic glycemic response to glucagon, shaped by GHD etiology.

Hypoglycemia (<60 mg/dL) occurred in 33 patients (18.3%) and more frequently in CCS (54.5%). Eleven subjects had values < 50 mg/dL between 90 and 150 minutes, with a minimum of 39 mg/dL in 1 I-GHD patient. No symptoms, adverse events, or test interruptions were reported.

### Association Between GH Peak to GST and BMI SDS

An inverse association between GH peak to GST and BMI SDS was observed in the whole cohort (Spearman's ρ = −0.457, *P* < .001). In order to evaluate the impact of the original diagnosis on this association, a multiple linear regression model was fitted, normalizing the GH peak to GST values by the natural logarithm transformation. After adjusting by original GHD diagnosis, separating nonidiopathic patients into OM- and OS-GHD, the regression coefficient β for BMI SDS dropped from −0.39 to −0.14 but remained statistically significant (*P* = .003). Similarly, when splitting nonidiopathic patients into congenital and acquired GHD, the corresponding coefficient was −0.17 (*P* < .001). Stratified analysis indicated that the association was stronger in nonidiopathic patients, even if the test for interaction in the multivariable regression model provided nonsignificant results (*P* = .301 for the model including OM-GHD and OS-GHD and *P* = .091 for the model including congenital and acquired GHD, respectively).

When CCS patients were excluded, the association between GH peak to GST and BMI SDS was reduced in both univariable (β = −0.22, *P* < .001) and, to an even greater extent, multivariable models (β = −0.098, *P* = .056 splitting nonidiopathic patients into OM and OS-GHD and β = −0.12, *P* = .022 splitting such patients into congenital and acquired GHD, respectively).

### Association Between IGF-1 SDS and BMI SDS

IGF-1 SDS showed a significant negative correlation with BMI SDS in the entire cohort. (ρ = −0.331, *P* < .0001). However, regression analysis showed that this effect was entirely explained by groups, rather than BMI SDS. Indeed, controlling for group effect, the association between BMI SDS and IGF-1 SDS was lost (*P* = .556 for the model including OM and OS GHD groups and *P* = .243 for the model including congenital and acquired GHD). Consistently, stratified analysis showed a similar flat trend for I-GHD, OM-GHD, and OS-GHD. Conversely, an increasing trend was observed for patients with congenital GHD, but the sample size for this group of patients was limited (n = 18), and statistically significance was not reached (*P* = .336, test for interaction).

## Discussion

The GST is increasingly recognized as a reliable alternative to ITT, particularly when ITT is contraindicated (33-36). Previous studies in adults suggest GH cutoffs between 1.0 and 3.0 μg/L after GST have high diagnostic accuracy, although optimal thresholds vary based on pituitary deficiency severity and glucagon dosing methods (36-42). However, its clinical adoption remains limited, with a European audit indicating GST utilization in only 6% of adult GHD assessments (34). Moreover, data on GSTs’ accuracy during the transitional period from childhood to adulthood are sparse, especially concerning factors such as BMI and GHD etiology.

In the current study, we analyzed GH secretion in response to GST in 180 adolescents and young adults with CO-GHD. Participants were categorized into 3 etiological groups: I-GHD, OM-GHD, and OS-GHD. Consistent with the existing literature, our analysis demonstrated a significant inverse relationship between BMI and peak GH secretion, reinforcing the inhibitory role of adiposity on GH release (40, 43-48). Indeed, BMI-adjusted GH cutoffs have been proposed previously to enhance diagnostic accuracy in patients with obesity for different stimulation tests (40, 44). A recent meta-analysis further supports this association, indicating approximately a 12% decrease in peak GH secretion for each unit increase in BMI z-score (49).

Obesity was notably prevalent among patients with organic forms of GHD, particularly in CSS. At retesting, 13.3% of our cohort had obesity, with the highest prevalence in the OS-GHD group (35.1%) and the lowest in the I-GHD group (5.0%). CCS, comprising 42% of our cohort, accounted for approximately two-thirds of obesity cases, predominantly among those with prior cranial irradiation, highlighting the strong relationship between persistent adiposity, hypothalamic-pituitary damage, and cancer treatment (50-52).

Using a GH peak cutoff of <5.8 µg/L, selected for its accuracy in diagnosing persistent GHD during transition (21), we confirmed persistent deficiency in 50.0% of the cohort. Persistence varied significantly by etiology and was highest in OS-GHD patients (91.9%), followed by OM-GHD (77.8%), and lowest in I-GHD cases (8.6%, *P* < .001). This gradient aligns with previous studies indicating higher persistence risk in organic GHD associated with brain tumors or radiation-induced damage (1, 15, 53-57). Current guidelines thus support omitting retesting in individuals at high risk, such as those with structural hypothalamic-pituitary abnormalities or multiple pituitary hormone deficiencies (5, 19, 33).

Analysis of GH dynamics during GST supported these observations, showing peak GH concentrations at approximately 150 minutes poststimulation across all groups. However, GH responses were significantly reduced in the OS-GHD and acquired GHD groups compared to I-GHD patients (*P* < .01), likely due to irreversible hypothalamic-pituitary damage (58, 59). Particularly, 93.8% of acquired GHD and 81.1% of OS-GHD patients were CSS, who also accounted for 66.7% of individuals with obesity. This overlap suggests that diminished GH responses in these groups may be influenced not only by structural damage but also by increased adiposity, supporting a potential pathophysiological link between impaired GH secretion and obesity in CCS (60). Indeed, it is worth noting that CCS exhibited significant lower GH peaks (median 1.38 µg/L) than non-CCS, highlighting substantial endocrine sequelae from tumor location and cranial therapies (60-62). Specifically, among the 16 CCS patients with obesity, hypothalamic involvement was common and attributable to various etiologies; 9 had received intracranial radiotherapy or total body irradiation, 4 had a history of craniopharyngioma, 2 were diagnosed with Langerhans cell histiocytosis, and 1 had a pilocytic astrocytoma. These conditions are all associated with a high risk of hypothalamic–pituitary disruption due to compression, infiltration, or treatment-related injury and are well-documented contributors to hypothalamic obesity in CCS (63). In particular, craniopharyngioma is well documented to result in multiple pituitary hormone deficiencies, with approximately half of patients developing obesity and eating disturbances following surgery (63, 64).

Although univariate analysis showed a significant inverse correlation between BMI SDS and GH peak, multivariate regression demonstrated that etiological group was the dominant predictor of GH response. Thus, while adiposity may modestly attenuate GH secretion (47, 65, 66), the underlying hypothalamic-pituitary pathology significantly outweighs its influence. Nevertheless, in the entire cohort, BMI SDS remained independently associated with GH peak after adjustment for etiology, indicating that adiposity contributes as an additional, although secondary, modulator of GH secretion.

When CCS were excluded, the model retained moderate explanatory power. Specifically, group differences remained significant, whereas the effect of BMI SDS did not reach statistical significance, suggesting that hypothalamic-pituitary damage, rather than adiposity, was the predominant determinant of GH response. In the CCS subgroup, OS-GHD status remained significantly associated with lower GH peak, while the effect of BMI SDS was no longer statistically significant, indicating a marginal influence of adiposity in this population.

Overall, our findings suggest that both the underlying hypothalamic–pituitary etiology and adiposity influence GH responsiveness to glucagon during the transition period. Although etiology exerted the strongest effect, the residual impact of BMI supports the concept that structural damage and adiposity may interact to blunt GH secretion. This interplay was particularly relevant in CCS, in whom hypothalamic–pituitary injury and excess weight frequently coexist. However, in this subgroup the severity of the structural damage largely overshadowed the modulatory influence of adiposity, rendering the independent effect of BMI no longer statistically detectable.

Several studies have reported an inverse correlation between BMI and GH response to GST, although results vary across populations. Gómez et al (39). observed this association in healthy controls but not in organic GHD, suggesting that hypothalamic–pituitary damage may mask adiposity's effects. Other studies confirmed a negative BMI–GH relationship (40, 42, 46, 67-70). Conversely, Berg et al (37) and Hamrahian et al (41) found no significant correlation, likely due to severe hypothalamic–pituitary impairment. Consistent with these mixed findings, our results indicate that BMI exerts only a modest effect on GH secretion, with etiology emerging as the dominant determinant.

Analysis of IGF-1 SDS and BMI SDS revealed a significant inverse correlation in the overall cohort (ρ = −0.331), which disappeared after adjusting for etiology, indicating that IGF-1 levels are mainly determined by underlying pituitary pathology rather than adiposity. Consistently, IGF-1 SDS was lowest in OS-GHD, reflecting more severe hypothalamic–pituitary dysfunction.

Similar findings were reported by Diri et al (42), who found no significant relationship between BMI and IGF-1 SDS across their entire cohort; however, after excluding patients with ≥3 pituitary hormone deficiencies, a significant inverse correlation between BMI and IGF-1 SDS emerged.

Although a low serum IGF-1 level is recognized as a strong marker of GHD, particularly in patients with multiple pituitary hormone deficiency, normal IGF-1 levels do not exclude GHD diagnosis (21). IGF-1 SDS alone has limited diagnostic accuracy in children and adolescents, reinforcing the importance of interpreting IGF-1 levels in conjunction with clinical features and GH stimulation test outcomes (71).

In our cohort, the GST showed an excellent safety profile, as no patient experienced significant adverse effects such as nausea, vomiting, or abdominal discomfort, which are the most commonly reported in previous studies. This aligns with evidence indicating that the glucagon test is generally well tolerated across age groups, with gastrointestinal symptoms variably reported in less than 10% to 34% of cases (37, 67, 68). The AACE guidelines (16) endorse the GST as a valid alternative to the ITT, emphasizing its favorable safety profile and lower risk of hypoglycemia. Although glucose fluctuations, including biochemical hypoglycemia, may occur, they are typically mild and asymptomatic, as observed in our study. Despite biochemical hypoglycemia in 18.3% of patients, mainly those with hypothalamic involvement, the GST was well tolerated in all cases, with no symptomatic events or test interruptions, confirming its feasibility even in vulnerable subgroups.

This study demonstrates that GST is a reliable diagnostic tool for assessing persistent GHD during the transition from adolescence to adulthood, particularly in individuals with organic etiologies.

Our results suggest that adopting lower GH cutoff thresholds in subjects with obesity during the transition phase, such as those recommended by the AACE (16), which propose different cutoffs after GST (≤3 and ≤1 µg/L, depending on pretest probability and BMI), may not be appropriate in this age group. These thresholds, although established for adults, have not yet been validated in adolescents and young adults undergoing transition, and their applicability in this age group remains uncertain. Moreover, it should be considered that glucagon has been shown to elicit a strong GH response in children, even in those with underlying hypothalamic–pituitary defects, suggesting a greater stimulatory potency compared to other provocative tests (72).

The strengths of our study include a large and well-characterized cohort, including a substantial proportion of CCS, which allowed for meaningful subgroup comparisons and the application of multivariable analyses.

Notably, the test proved safe across the spectrum of disease severity; despite biochemical hypoglycemia in 18.3% of subjects, no clinically significant events or test interruptions occurred. Together, these observations reinforce current international guidance that the GST is a thorough alternative when the ITT is contraindicated. Nonetheless, certain limitations should be acknowledged. The retrospective design inherently introduces potential selection and information biases. Moreover, since not all patients underwent an ITT, we were unable to compare GST results with the diagnostic gold standard or explore the applicability of BMI-adjusted GH cutoff values.

Another methodological consideration is the glucagon dosing regimen used in the GST. In our study, we administered a fixed intramuscular dose of 1 mg of glucagon, in line with commonly used adult protocols. Although the weight-based glucagon regimen has been shown to correlate with the ITT in assessing GH secretion in children (72), neither dosing approach has been systematically evaluated in the transition age group. In adults, there is still no consensus on the optimal glucagon dosing strategy, as both fixed-dose and weight-based protocols are used in clinical practice. Fixed-dose regimens usually consist of 1 mg intramuscular (1.5 mg if >90 kg), whereas weight-based protocols use 0.03 mg/kg. Yuen et al (67) applied both dosing strategies and found an inverse correlation between peak GH and BMI, which was more pronounced with the weight-based regimen. Conversely, Hamrahian et al (41), using the same fixed (1-1.5 mg) and weight-based (0.03 mg/kg, mean 2.4 mg) doses, found no significant association between peak GH and BMI, even after excluding patients with multiple pituitary deficits. Although available data are inconclusive, BMI variability might have influenced GH response; however, using a 1 mg fixed dose remains a reasonable approach in transition-age patients.

In addition, the relatively small number of patients in the OS-GHD group may reduce the statistical power of subgroup analyses within this high-risk population. Moreover, our reliance on BMI SDS as a proxy for adiposity represents a limitation, as it does not capture the complexity of body composition or fat distribution, and no anthropometric measures for estimating fat mass or central adiposity (such as waist circumference or waist-to-height ratio) were available in our cohort. Future studies incorporating more precise assessments of body composition, including dual-energy X-ray absorptiometry-derived parameters and evaluation of visceral adiposity, could provide a clearer understanding of the relationship between adiposity and GH secretion, particularly in CCS, and especially those exposed to cranial or total body irradiation, who are prone to increased visceral fat accumulation and higher metabolic risk (73-75). Potter et al proposed clinically relevant percent body fat thresholds for overweight, and obesity based on metabolic risk, emphasizing the limitations of BMI in accurately reflecting adiposity (76). Similarly, Dichtel et al (40) identified a modest, independent association between visceral adipose tissue and reduced GH response, although this effect was attenuated after adjusting for BMI, suggesting that specific fat depots may influence somatotropic function beyond general adiposity.

In conclusion, this study highlights the GST as a reliable and effective diagnostic tool for assessing persistent GHD during the transition from adolescence to adulthood, especially in individuals with organic or acquired GHD etiologies, such as CCS. The magnitude of the GH response is primarily driven by the nature of the underlying hypothalamic-pituitary damage, with CCS and those with cranial irradiation showing the most significant GH deficits and highest likelihood of persistent GHD.

While an inverse relationship between BMI and GH secretion was observed, the multivariate analysis revealed that adiposity acted as a secondary factor, with structural hypothalamic-pituitary damage being the dominant determinant of GH response. These findings challenge the applicability of adult BMI-based GH cutoffs during the transition phase and support the need for transition-specific diagnostic thresholds. Moreover, the study supports the safety and feasibility of the GST in individuals with varying degrees of hypothalamic-pituitary dysfunction, reinforcing its utility as a valuable alternative to the ITT. The study also cautions against using lower GH cutoffs for obese adolescents, as recommended for adults, as these thresholds have not been validated for this population. Future prospective studies focusing on more accurate body composition measures and standardized glucagon dosing protocols will be essential to further refine diagnostic cutoffs and elucidate the true impact of adiposity on GH secretion in the transition period.

## Data Availability

The data sets produced through the current study are not publicly available but are available from the corresponding author on reasonable request.
